# New Professors in Harvard University

**Published:** 1847-06

**Authors:** 


*New Professors in Harvard University.*—We copy the following from the Boston Medical and Surgical Journal. The worthiness and wisdom of the appointments will be appreciated more widely than by the medical profession of Massachusetts alone. We tender our congratulations not only to the appointees, but to our Alma Mater.

We doubt not that the labors of the new professors will redound to their own honor, and to that of the institution.

"We learn that the Corporation of Harvard University, since the resignation of Dr. Warren, have appointed three new professors, two of whom are to be attached to the Massachusetts Medical College in Boston, and one to the University at Cambridge. The new incumbents are, Oliver W. Holmes, M. D., Professor of Anatomy and Physiology; John B. S. Jackson, M. D., Professor of Pothological Anatomy, and Curator; and Jeffries Wyman, M. D., Hersey Professor of Anatomy at Cambridge.

All the above gentlemen are well known to the public as working men, who by their own talents and persevering industry, have raised themselves to the head of the departments in science which they are respectively to teach. Dr. Holmes is eminently distinguiihed in our medical community as a talented and eloquent lecturer, and an accomplished scholar and man of science. He has been for ten years a successful teacher of Anatomy and Physiology in the Tremont Street Medical School, and was for two years a professor of the same branches in Dartmouth College. Dr. Jackson, as a Pathological Anatomist, has no superior in the United States. The medical profession owe him much for many years of unrequited labor, which nothing but an ardent devotion to science for its own sake, could have sustained. The rich collection of morbid anatomy of the Society for Medical Improvement, and its forthcoming descriptive catalogue, are mainly indebted to him for their existence. Dr. Wyman is our best comparative anatomist, and has filled with great credit the Chair of Professor of Anatomy in Richmond, Va., for the last two years. He will form an important acqusition to the new scientific school about to be established at Cambridge. We are gratified that the choice of the Corporation has fallen on candidates in whose favor we believe the general suffrage of the medical profession in Massachusetts would have cordially united."

